# Decisional preferences and distress among kidney transplant recipients with impaired graft function

**DOI:** 10.3389/ti.2026.16268

**Published:** 2026-06-01

**Authors:** Bilgin Osmanodja, Jakob Joachim Spencker, Ömer Ege Ömeroğlu, Zeineb Sassi, Sascha Eickmann, Roland Roller, Aljoscha Burchardt, Michael Hahn, Tabea Ott, Peter Dabrock, Sebastian Möller, Klemens Budde, Anne Herrmann

**Affiliations:** 1 Department of Nephrology and Medical Intensive Care, Charité - Universitätsmedizin Berlin, Berlin, Germany; 2 Department of Epidemiology and Preventive Medicine, Universität Regensburg, Regensburg, Germany; 3 German Research Center for Artificial Intelligence, Berlin, Germany; 4 Institute for Systematic Theology, Friedrich-Alexander-Universität Erlangen-Nürnberg, Erlangen, Germany; 5 Department of Systematic Theology, University of Vienna, Vienna, Austria; 6 Quality and Usability Lab, Technical University of Berlin, Berlin, Germany; 7 Department of Internal Medicine III, University Hospital Regensburg, Regensburg, Germany; 8 Bavarian Cancer Research Center, Regensburg, Germany

**Keywords:** graft loss, kidney transplantation, patien-centered care, patient-reported outcome measure (PROM), shared decision making (SDM)

Dear Editors,

After transplantation, 57% of kidney transplant recipients (KTR) experience graft loss, while 43% die with a functioning graft [1], Supplementary References S1, S2. When graft loss occurs, KTR face preference-sensitive decisions similar to those in advanced chronic kidney disease (CKD) and kidney failure (KF) [2], Supplementary Reference S3. In CKD and KF, implementing shared decision-making (SDM) improves satisfaction with the selected kidney replacement therapy [3], Supplementary References S4, S6. For KTR approaching graft loss, however, evidence-based SDM interventions are lacking. In conventional physician-centered care, reported rates of conversations about treatment options after graft loss are as low as 13%, and the associated SDM process has not been studied [4]. The KDIGO (Kidney Disease: Improving Global Outcomes) guideline “Challenges in the management of the kidney allograft: from decline to failure” underscores the importance of SDM and advance care planning in this phase while also highlighting the limited evidence base [2].

The “Prospectively investigating the Impact of AI on Shared Decision-Making in Post-Kidney Transplant Care” (PRIMA-AI; NCT06056518) study investigates whether implementing an AI-based model predicting 1-year risk of graft loss increases the frequency of conversations about kidney replacement therapy in case of graft loss and supports shared decision-making (SDM) as graft function declines. In this research letter, we report patients’ self-reported decisional preferences, their decisional experiences, and patient-reported outcome measures (PROMs) including distress [5], Supplementary References S7–S9. This sequential, longitudinal mixed-methods study was nested within the randomized, single-center PRIMA-AI study comparing the AI-supported approach with usual care. We enrolled 76 German-proficient KTRs with eGFR <30 mL/min/1.73 m^2^ from two outpatient transplant clinics. Recruitment was terminated early (planned n = 122) due to recruitment difficulties. We summarize quantitative survey data for the full cohort from the baseline visit. The ethics committee of Charité - Universitätsmedizin Berlin approved the study and detailed protocols have been published previously [6], Supplementary Reference S11. Full methods are provided in the Supplementary Material.

From January 19, 2024 to September 18, 2024, 76 KTRs (age: 58.6 ± 17 years; transplant age: 14.4 ± 7.5 years) were enrolled, 32% (24/76) of which were female. Most participants reported German nationality (93%, 71/76), as well as German origin and mother tongue (89%, 68/76). Educational attainment was high: 65.8% (50/76) reported completing high school or higher; 50% (38/76) reported a job qualification; and 39.4% (30/76) reported a university degree or comparable qualification (Detailed demographics: Supplementary Table S1, CONSORT flowchart: Supplementary Figure S1).

Preferred decision-making roles were assessed using the validated Control Preferences Scale (CPS) with the question: “How would you like to decide on kidney replacement therapy after graft loss?”. Of 76 enrolled patients, 71 (93%) provided valid baseline CPS data. At baseline, 48% (34/71) preferred an active role, 44% (31/71) a collaborative role, and 8.5% (6/71) a passive (paternalistic) role (Supplementary Table S2). A minority of 33% (25/76) already provided information about their decision-making experiences regarding graft loss at the baseline visit. They reported a higher proportion of passive decision-making experiences compared to the overall decision-making preferences (28% vs. 8.5%, Figure 1).

**FIGURE 1:**
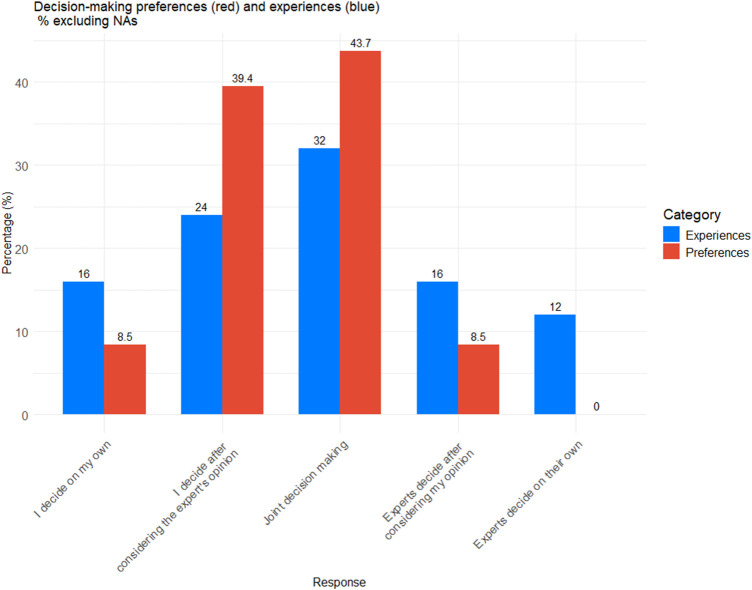
Decision-making preferences (red) of 71/76 participants assessed with the Control Preferences Scale (CPS), and decision-making experiences (blue) of 25/76 participants assessed with the CPS post questionnaire show that patients experienced passive decision-making roles more often than the overall cohort preferred those.

From baseline PROMs, we report self-rated general health and the Distress Thermometer (DT), a brief, intuitive, and cross-culturally validated measure of psychological distress (0 = no distress to 10 = extreme distress). General health was rated as bad by 7.9% (6/76), mediocre by 42.1% (32/76), good by 39.5% (30/76), and very good by 1.3% (1/76), with 9.2% (7/76) missing. Median distress in the past week was 6 (interquartile range 4–7) (Supplementary Figure S2). The following reasons for distress were reported in >30% of the surveys: exhaustion (61.9%), sleep (51.6%), pain (48.2%), concerns (46.2%), mobility (43.8%), dry/itching skin (35.5%), diarrhea (35.0%), digestive problems (32.3%), sadness (31.2%), and fears (30.3%).

Most KTRs with impaired graft function preferred an active or collaborative role when considering kidney replacement therapy after potential graft loss. Decisional preferences resemble patterns seen in other serious illnesses such as cancer and heart disease [7], Supplementary References S12, S13. In the baseline surveys, a higher proportion of patients reported experiencing a passive role than was preferred in the overall cohort. Together with previously reported low conversation rates in standard care, this supports prioritizing interventions that reliably enable SDM as graft function deteriorates. One promising approach are patient decision aids, which provide clear, comprehensive information on risks and benefits and can be delivered as booklets or web-based tools [3, 8]. They improve patient knowledge, risk perception, and decisional conflict related to feeling uninformed [3, 8]. Since KTR at risk for graft loss face comparable decisions as patients with CKD and KF, adapting validated decision aids from those settings seems sensible [3]. Importantly, consultation length is generally unchanged when decision aids are used beforehand and increases by only 1.5 min when used during a visit [8].

We also observed substantial distress in this cohort, comparable to levels reported in patients receiving chemotherapy. In oncology, DT thresholds of 4-5 are commonly used to indicate clinically significant distress, and a recent meta-analysis suggests that around half of lung cancer patients meet such criteria [9], Supplementary Reference S9. Importantly, the reasons for distress were physical symptoms above all, with almost half of the patients reporting pain, which as a manageable symptom should deserve more attention in our practice. While these findings should be confirmed in multi-center observational studies including patients with more diverse backgrounds and all levels of graft function, our findings still warrant broader routine assessment of distress in post-transplant care. A systematic evaluation for physical symptoms and clearer pathways to psychological and social support is needed when distress is high. There is a need for evidence-based, easy-to-adopt guidelines and strategies on how to increase the uptake of such offers in KTR. For example, individually tailored referrals to supportive care programmes, including social prescriptions, may deliver accessible information and support to address unmet psychosocial and practical needs Supplementary Reference S14. This may improve both patients’ and their support persons’ health and wellbeing and enhance multidisciplinary collaboration among various healthcare providers.

Limitations include the single-center and non-observational design both of which resulted in a relatively small sample size. Given the inclusion criteria such as German-fluency and the topic of the trial (AI-based risk prediction), we assume that there was considerable participation bias toward participants of German origin and higher socioeconomic status than the general transplant population.

## Data Availability

The raw data supporting the conclusions of this article will be made available by the authors, without undue reservation.
