# Supplementary material for: Decisional preferences and distress among kidney transplant recipients with impaired graft function
Source: Transpl Int. 2026 Jun 1;39:16268. doi: 10.3389/ti.2026.16268 (PMC13265414; doi:10.3389/ti.2026.16268)
Supplement: Supplementary file 1 [file DataSheet1.pdf]

Supplementary Material to the Research Letter *"Decisional preferences and distress among kidney transplant recipients with impaired graft function"*

Capsule sentence summary (31 words)

Kidney transplant recipients facing graft loss prefer shared decision-making but often experience passive roles. Distress levels were comparable to those reported in cancer patients, warranting routine distress screening in post-transplant care.

Supplementary Methods

Study Overview

In the PRIMA-AI study, a machine-learning based risk prediction model predicting the risk of graft loss within the next year is incorporated into the proprietary electronic health record (EHR) at our kidney transplant center.[S5,S15] Patients are randomized 1:1 into usual care or the additional use of the risk prediction and physicians are free to use these risk scores in their consultations for patients in the intervention group.[6,S11] The primary outcome is conversation frequency about the treatment options after graft loss. Secondary outcomes include medical endpoints such as the proportion of patients requiring dialysis within the study period, the respective dialysis modality and dialysis access chosen and the need for emergency dialysis initiation. Other secondary endpoints include scores that capture the associated SDM process (control preferences scale and post-decision control preferences scale [CPS-post], collaboRATE mean and top score) and quality of life measures including patient distress using the distress thermometer.[5,S7-S9] Surveys examining the primary outcome, decisional quality, control preferences, and other secondary outcomes are assessed at baseline and every three months until 12 months after randomization.[6] Qualitative interviews are conducted with clinicians (nephrologists and nephrology residents) and with all patients in the intervention group as well as purposely sampled patients in the control group with treatment decisions.[S11]

The recruitment was terminated before the planned sample size of 122 patients was reached due to recruitment difficulties. However, the achieved sample size was deemed enough to achieve theoretical saturation in the qualitative analyses of the study. For the present study, quantitative data from the baseline surveys for control preferences scale (CPS) are reported. The ethics committee of Charité - Universitätsmedizin Berlin approved this study.

Data collection

Self-reported demographic data and decisional quality measures are collected using in-person surveys at baseline, and at 3 to 6-month follow-up; eGFR is extracted from medical records. We used the Control Preferences Scale (CPS), a validated, single-item, 5-point scale to indicate the degree of control patients desire in major healthcare decisions.[5] The measure categorizes patients into three roles that indicate their preference for decision-making: active patients prefer to be the sole or primary decision-maker with clinician input; collaborative patients prefer to more equally share decision-making with clinicians. Finally, passive patients prefer clinicians to be the primary decision-maker, considering patient values. In this study, the CPS was specified to concern the decision-making around the kidney replacement therapy after graft loss.

Statistical analysis

We report absolute and relative frequencies, mean ± standard deviation for normally distributed variables or median (interquartile range) for other variables. For correlation of preferred and actual decision making preferences, we use the 5-point CPS and calculate repeated measures correlation assuming that all survey from one patient are repeated measures as implemented in the R-package rmcorr.[S16] To calculate the mean difference between preferred and actual decision making preferences, we first calculated a mean difference for every patient (excluding missing values) and afterwards calculated an overall mean +/- standard deviation. No imputation methods were used. All statistical analyses were performed using R, version 4.5.1.[S17]

Supplementary Table S1. Baseline characteristics of the study cohort as mean ± standard deviation or relative (absolute) frequency in %. IB, International Baccalaureate;

	Baseline demographics	N = 76
Patient age in years		58.64 ± 17.01
Time since last transplantation in years		14.42 ± 7.53
Time since first kidney replacement therapy		18.24 ± 7.32
Sex	- male<br>- female	68% (52)<br>32% (24)
eGFR (ml/min/1.73m^2)		26.0 ± 8.24
Treatment group	- intervention<br>- control	53% (40)<br>47% (36)
Nationality/Origin	- German nationality<br>- Born in Germany<br>- Mother tongue German<br>- both parents born in Germany<br>- no answer	71 (93.4%)<br>68 (89.5%)<br>68 (89.5%)<br>61 (80.3%)<br>4 (5.3%)
Personal status	- married<br>- civil partnership<br>- unmarried<br>- widowed/divorced<br>- no answer	49 (64.4%)<br>7 (9.2%)<br>11 (14.5%)<br>5 (6.6%)<br>4 (5.3%)
Highest school degree	- none<br>- lower secondary school diploma<br>- high school diploma<br>- (Fach-)abitur (IB)<br>- other/no answer	6 (7.9%)<br>12 (15.8%)<br>19 (25%)<br>31 (40.8%)<br>8 (10.5%)
Highest job qualification	- job training completed<br>- university degree/master's certificate<br>- none<br>- no answer	38 (50%)<br>30 (39.4%)<br>4 (5.3%)<br>4 (5.3%)

Netto household income	- < 500 €<br>- 500-999€<br>- 1000 - 1499 €<br>- 1500 - 1999 €<br>- 2000 - 2499 €<br>- 2500 - 2999 €<br>- 3000 - 3499 €<br>- ≥ 3500 €<br>- no answer	2 (2.6%)<br>6 (7.9%)<br>9 (11.8%)<br>6 (7.9%)<br>10 (13.2%)<br>6 (7.9%)<br>8 (10.5%)<br>22 (28.9%)<br>7 (9.2%)

Supplementary Table S2. Absolute and relative frequencies of decision-making preferences among kidney transplant recipient over time using the control preferences scale (3-point and 5-point).

**Preferred Role**	**Item Description, n (%)**	**Baseline (n=71)**
Active	I prefer to make the final selection about which renal replacement therapy I will receive	6 (8.5%)
	I prefer to make the final selection of my renal replacement therapy after seriously considering my doctor's opinion	28 (39.4%)
Collaborative	I prefer that my doctor and I share responsibility for deciding which renal replacement therapy is best for me	31 (44.6%)
Passive	I prefer that my doctor makes the final decision about which renal replacement therapy will be used, but seriously considers my opinion	6 (8.5%)
	I prefer to leave all decisions regarding renal replacement therapy to my doctor	0 (0%)

Supplementary Figures

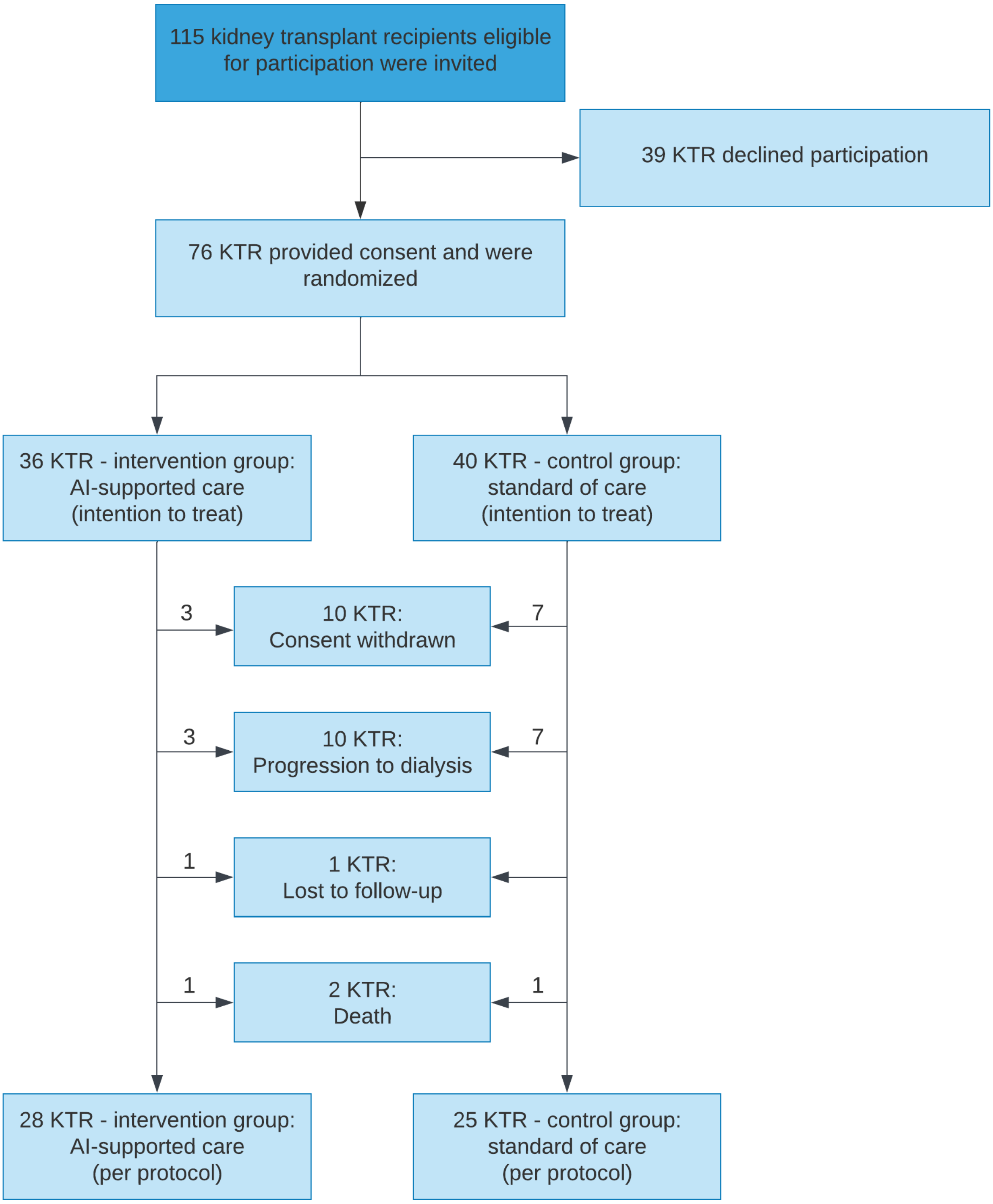

Supplementary Figure S1. CONSORT flow chart.

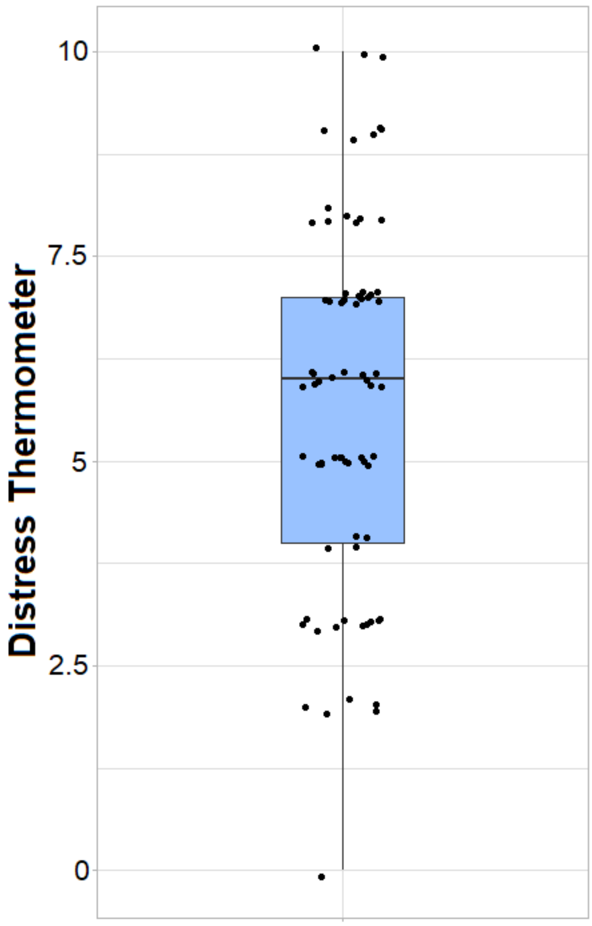

Supplementary Figure 2. Distress thermometer from baseline surveys displaying the grade of self-reported distress from 0 (no distress) to 10 (extreme distress) within the last week in kidney transplant recipients with impaired graft function.

Supplementary References

S1. Mayrdorfer M, Liefeldt L, Osmanodja B, Naik MG, Schmidt D, Duettmann W, Hammett C, Schrezenmeier E, Friedersdorff F, Wu K, Halleck F, Budde K. A single centre in-depth analysis of death with a functioning kidney graft and reasons for overall graft failure. Nephrol Dial Transplant. 2023 Jul 31;38(8):1857-1866. doi: 10.1093/ndt/gfac327. PMID: 36477607; PMCID: PMC10387383.
S2. Ying T, Shi B, Kelly PJ, Pilmore H, Clayton PA, Chadban SJ. Death after Kidney Transplantation: An Analysis by Era and Time Post-Transplant. J Am Soc Nephrol. 2020 Dec;31(12):2887-2899. doi: 10.1681/ASN.2020050566. Epub 2020 Sep 9. PMID: 32908001; PMCID: PMC7790214.
S3. Sellars M, Clayton JM, Morton RL, Luckett T, Silvester W, Spencer L, Pollock CA, Walker RG, Kerr PG, Tong A. An Interview Study of Patient and Caregiver Perspectives on Advance Care Planning in ESRD. Am J Kidney Dis. 2018 Feb;71(2):216-224. doi: 10.1053/j.ajkd.2017.07.021. Epub 2017 Nov 11. PMID: 29132946.
S4. Yu X, Nakayama M, Wu MS, Kim YL, Mushahar L, Szeto CC, et al. Shared decision-making for a dialysis modality. Kidney Int Rep. 2022;7(1):15-27.
S5. Kanbay M, Basile C, Battaglia Y, Mantovani A, Yavuz F, Pizzarelli F, et al. Shared decision making in patients with kidney failure. Nephrol Dial Transplant. 2023.:gfad211.

S6. Murea M, Grey CR, Lok CE. Shared decision-making in hemodialysis vascular access practice. Kidney Int. 2021;100(4):799-808.
S7. Elwyn G, Barr PJ, Grande SW, Thompson R, Walsh T, Ozanne EM. Developing CollaboRATE: a fast and frugal patient-reported measure of shared decision making in clinical encounters. Patient Educ Couns. 2013 Oct;93(1):102-7. doi: 10.1016/j.pec.2013.05.009. Epub 2013 Jun 12. PMID: 23768763.
S8. Forcino RC, Barr PJ, O'Malley AJ, Arend R, Castaldo MG, Ozanne EM, Percac-Lima S, Stults CD, Tai-Seale M, Thompson R, Elwyn G. Using CollaboRATE, a brief patient-reported measure of shared decision making: Results from three clinical settings in the United States. Health Expect. 2018 Feb;21(1):82-89. doi: 10.1111/hex.12588. Epub 2017 Jul 5. PMID: 28678426; PMCID: PMC5750739.
S9. Donovan KA, Grassi L, McGinty HL, Jacobsen PB. Validation of the distress thermometer worldwide: state of the science. Psychooncology. 2014 Mar;23(3):241-50. doi: 10.1002/pon.3430. Epub 2013 Nov 11. PMID: 25160838.
S10. Roller R, Mayrdorfer M, Duettmann W, Naik MG, Schmidt D, Halleck F, Hummel P, Burchardt A, Möller S, Dabrock P, Osmanodja B, Budde K. Evaluation of a clinical decision support system for detection of patients at risk after kidney transplantation. Front Public Health. 2022 Oct 25;10:979448. doi: 10.3389/fpubh.2022.979448. PMID: 36388342; PMCID: PMC9641169.
S11. Sassi Z, Eickmann S, Roller R, Osmanodja B, Burchardt A, Samhammer D, Dabrock P, Möller S, Budde K, Herrmann A. Prospectively investigating the impact of AI onshared decision-making in post kidney transplant care (PRIMA-AI): protocol for a longitudinal qualitative study among patients, their support persons and treating physicians at a tertiary care centre. BMJ Open. 2024 Oct 1;14(10):e081318. doi: 10.1136/bmjopen-2023-081318. PMID: 39353696; PMCID: PMC11448240.
S12. Cousino, Melissa K., et al. "Medical and end-of-life decision-making preferences in adolescents and young adults with advanced heart disease and their parents." *JAMA Network Open* 6.5 (2023): e2311957-e2311957.
S13. Tariman JD, Berry DL, Cochrane B, Doorenbos A, Schepp K. Preferred and actual participation roles during health care decision making in persons with cancer: a systematic review. Ann Oncol. 2010 Jun;21(6):1145-1151. doi: 10.1093/annonc/mdp534. Epub 2009 Nov 25. PMID: 19940010; PMCID: PMC4200024.
S14. Husk, Kerryn, et al. "What approaches to social prescribing work, for whom, and in what circumstances? A realist review." Health & social care in the community 28.2 (2020): 309-324.
S15. Schmidt D, Osmanodja B, Pfefferkorn M, Graf V, Raschke D, Duettmann W, Naik MG, Gethmann CJ, Mayrdorfer M, Halleck F, Liefeldt L, Glander P, Staeck O, Mallach M, Peuker M, Budde K. TBase - an Integrated Electronic Health Record and Research Database for Kidney Transplant Recipients. J Vis Exp. 2021 Apr 13;(170). doi: 10.3791/61971. PMID: 33938875.
S16. Bakdash JZ, Marusich LR. Repeated Measures Correlation. Front Psychol. 2017 Apr 7;8:456. doi: 10.3389/fpsyg.2017.00456. Erratum in: Front Psychol. 2019 May 28;10:1201. doi: 10.3389/fpsyg.2019.01201. PMID: 28439244; PMCID: PMC5383908.
S17. R Core Team (2025). R: A Language and Environment for Statistical Computing. R Foundation for Statistical Computing, Vienna, Austria. https://www.R-project.org/.
